# Evaluating the quality of care for postpartum hemorrhage with a new quantitative tool: a population-based study

**DOI:** 10.1038/s41598-022-23201-0

**Published:** 2022-11-03

**Authors:** Hélène Didelot, François Goffinet, Aurélien Seco, Catherine Deneux-Tharaux, The EPIMOMS (EPIdémiologie de la MOrbidité Maternelle Sévère) Study Group, Elie Azria, Nathalie Baunot, Gaël Beucher, Marie-Pierre Bonnet, Marie-Hélène Bouvier-Colle, Lionel Carbillon, Anne Chantry, Coralie Chiesa-Dubruille, Catherine Crenn-Hebert, Catherine Deneux-Tharaux, Corinne Dupont, Jeanne Fresson, Gilles Kayem, Bruno Langer, Alexandre Mignon, Patrick Rozenberg, René-Charles Rudigoz, Aurélien Seco, Sandrine Touzet, Françoise Vendittelli

**Affiliations:** 1 Université Paris Cité, Obstetrical, Perinatal and Pediatric Epidemiology (Epopé) Research Team, Center for Epidemiology and Statistics (CRESS), INSERM U1153, Maternité Port Royal, 53 avenue de l’observatoire, 75014 Paris, France; 2 Department of Obstetrics and Gynecology, Cochin, Port-Royal Hospital, Assistance Publique-Hôpitaux de Paris, Paris, France; 3 URC-CIC Necker-Cochin, AP-HP, Paris, France; 4 Ile de France Paris Nord Perinatal Network, Paris, France; 5 Basse-Normandie Perinatal Network, Caen, France; 6 Inserm Epopé Research Team, CRESS, Paris, France; 7 Ile de France Naitre dans l’Est Francilien Perinatal Network, Bondy, France; 8 Ile de France 92 Nord Perinatal Network, Colombes, France; 9 Rhône-Alpes Aurore Perinatal Network, Lyon, France; 10 Lorraine Perinatal Network, Nancy, France; 11 Naitre en Alsace Perinatal Network, Strasbourg, France; 12 Société Française d’Anesthésie Réanimation, Paris, France; 13 Ile de France MYPA Perinatal Network, Poissy, France; 14 Santé Publique Pole, Hospices Civils de Lyon, Lyon, France; 15 Auvergne Perinatal Network, Clermont-Ferrand, France

**Keywords:** Health care, Public health, Epidemiology

## Abstract

To develop a new tool to assess the global quality of care for post-partum hemorrhage (PPH)—the leading preventable cause of maternal mortality worldwide—and to identify characteristics of maternity units associated with inadequate PPH management. This is a secondary analysis of the EPIMOMS population-based study conducted in 2012–2013 in 119 french maternity units (182,309 women who gave birth). We included women with severe PPH. We first developed a score to quantify the quality of care for PPH. Then, we identified characteristics of the maternity units associated with “inadequate care” defined by a score below the 25th percentile, with multi-level logistic regression adjusted for individual characteristics. The score combined 8 key components of care and took into account delivery mode and PPH cause. For PPH after vaginal delivery, the risk of inadequate care was increased in low versus high-volume maternity units (< 1000 deliveries/year: aOR-2.20 [1.12–4.32], [1000–2000 [deliveries/year: aOR-1.90 [1.02–3.56] compared to ≥ 3500 deliveries/year), in private versus public units (aOR-1.72 [1.00–2.97]), and in low versus high-level of care units (aOR-2.04 [1.24–3.35]). For PPH after cesarean, the only characteristic associated with an increased risk of inadequate care was the absence of 24/24-onsite anesthesiologist (aOR-4.34 [1.41–13.31]). These results indicate where opportunities for improvement are the greatest.

## Introduction

Postpartum hemorrhage (PPH) complicates about 10% of deliveries and is a leading cause of maternal mortality and severe morbidity worldwide^1–3^. The prevention and initial management of PPH are determinant in limiting progression to a severe form. Certain inappropriate or missing practices are clearly associated with a risk of worsening of the initial hemorrhage^4,5^. Given the large preventabiliy^6–8^ of maternal mortality and severe morbidity due to PPH, involving inadequate care^9–12^, there has been a proliferation of initiatives to improve practices in the management of PPH. The World Health Organization (WHO) and numerous countries have issued evidence-based guidelines^13–15^ accompanied by operational initiatives to expedite their dissemination and application^16–18^. Despite these efforts, the implementation of the guidelines into clinical practice remains insufficient and there is a persistent high rate of nonoptimal management of PPH^19–21^, so it is important to further characterize opportunities for improvement.

To properly identify non optimal management of PPH, we first need to dispose of an appropriate measurement tool to assess the global quality of care for PPH. Indeed, tools have been proposed, but have limitations. Global qualitative assessment by experts is subjective and therefore of questionable reproducibility^22^. Measurement of the implementation of a particular recommended item of management is more objective, but does not allow assessment of management as a whole^23–25^. So, there is a pressing need for a tool enabling the objective assessment of the global quality of care for PPH. Such a tool could be used to monitor the impact of practice improvement interventions or to make comparisons between hospitals.

Available literature suggests that the organizational environment of delivery care, approached by maternity hospitals’ characteristics, may influence the quality of obstetrics care^26^. Better understanding the degree to which PPH guidelines are integrated into practice and the impact of maternity hospitals characteristics on this integration, using an appropriate assessment tool, would enable identification of the units where the scope for improvement is greatest.

Our aims were, first, to develop a new quantitative tool to assess the global quality of care for first line management of PPH and, second, to identify the characteristics of maternity units associated with inadequate care.

## Materials and methods

### Population

This was a secondary analysis of data from the EPIMOMS prospective population-based study designed to study severe maternal morbidity^27,28^. The source population comprised 182,309 women who gave birth in the 119 maternity units of 6 regions between May 2012 and November 2013, i.e. approximately one-fifth of births in France over this period. The characteristics of the women who gave birth and of the maternity units were similar to those of the national profile^29^. All women included gave their consents for the use of their medical records. Of these women, 2540 (1.4%) presented severe maternal morbidity and were prospectively identified by the health professionals using a multi-criteria definition previously standardized by the Delphi expert consensus method at a national level (Appendix S2). The most severe acute maternal morbidity events were defined as “near-misses” according to the WHO definition^30^. Data on the women’s characteristics were collected using a questionnaire completed from a manual review of medical files by research midwives. A specific section on the management of PPH was provided for the women who presented severe PPH. The questionnaire contained precise information on the procedures implemented, the treatments administered, and the different timings. Data on the characteristics and organization of the maternity units (general organization, equipment, human resources) were collected in a maternity unit-specific questionnaire completed by the head of each maternity unit.

For our analysis, we first selected from the EPIMOMS women with severe maternal morbidity those whose morbidity was caused by severe PPH (N = 1580). These were women who presented PPH with at least one criterion of major bleeding (blood loss ≥ 1500 mL or transfusion ≥ 4 RBC units, surgical procedure/embolization) or PPH that led to acute hematologic dysfunction (anemia ≤ 7 g/dL, thrombocytopenia ≤ 50,000 platelets/mm^3^, disseminated intravascular coagulation), any organ failure according to EPIMOMS criteria, admission to an intensive care unit, or maternal death. We excluded some cases of PPH whose management was specific and not addressed in the French guidelines for PPH^31^: surgical wound without associated atony (N = 132), abnormal placental insertion (N = 112), uterine rupture (N = 36), amniotic fluid embolism (N = 13), vaginal thrombus without associated atony (N = 12), uterine inversion (N = 2), secondary PPH (N = 47), and PPH occurring at home (N = 7).

Of the 1233 women eligible, 129 had missing data on the score criteria and could not be included, so our study population finally included 1104 women with severe PPH (Appendix S3 shows the characteristics of the 129 women with missing scores). The flow chart is presented in Appendix S4.

### Outcome

We first developed a new quantitative tool to assess the global quality of care for PPH. The assessment score was based on key components of current guidelines selected by an independent and multidisciplinary panel of experts. All the experts had participated in the elaboration of the French guidelines for PPH^31^. Although those guidelines were edited in 2014 and thus posterior to the inclusion period, first line treatment for PPH management did not differ from the previous version in 2004^32^. Key components of PPH care were discussed during a first meeting and then validated by consensus during a second meeting. The 8 criteria selected targeted prevention and first-line treatment, i.e. care administered before any invasive procedure. When relevant, the criteria were specific to a mode of delivery or PPH etiology. Each criterion was weighted as major (weight of 2) or minor (weight of 1) according to the consensus opinion of the experts. For each woman, the quality score was calculated by combining these weighted criteria into a single figure, as the ratio of the number of criteria met (numerator) over the number of expected criteria (denominator). The calculations are detailed in Table 1. This score therefore corresponds to the percentage of expected items of management that were implemented adequately. The score ranged between 0% for a woman in whom none of the expected items of care were implemented adequately to 100% for a woman in whom all the expected items of care were implemented adequately.

The outcome for the analysis of determinants was inadequate care, a binary variable defined by an quality score strictly below the 25th percentile of distribution in the study population.

**Table 1:** Criteria of quality of care for PPH selected by the expert panel and calculation of the quality score.

Criteria^a^	Clinical context			
	Cesarean		Vaginal delivery	
	No uterine atony	Uterine atony	No uterine atony	Uterine atony
**Prophylactic** **uterotonic** **for** **the** **third** **stage** **of** **labor**				
Done	2	2	2	2
Not done	0	0	0	0
**Blood** **loss** **estimation** **written** **in** **the** **medical** **chart**				
Done	2	2	2	2
Not done	0	0	0	0
**First-line** **uterotonic**				
Administered ≤ 30 min^b^	2	2	2	2
Administered > 30 min^b^	1	1	1	1
Not administered	0	0	0	0
**Measurement** **of** **blood** **hemoglobin**^c^				
Done ≤ 60 min^b^	2	2	2	2
Not done or done > 60 min^b^	0	0	0	0
**Measurement** **of** **hemostatic** **function**^**||**^				
Done ≤ 60 min^b^	2	2	2	2
Not done or done > 60 min^b^	0	0	0	0
**Manual** **examination** **of** **the** **uterus**				
Done ≤ 30 min^b^	NA	NA	2	2
Not done or done > 30 min^b^	NA	NA	0	0
**Examination** **of** **the** **cervix** **and** **vagina**				
Done	NA	NA	1	1
Not done	NA	NA	0	0
**Second-line** **uterotonic** **(sulprostone)**				
Administered ≤ 30 min after first line uterotonic (oxytocin)	NA	2	NA	2
Administered (whatever the timing)	1	0	1	0
Not administered	0	0	0	0
Total: maximal number of points	11	12	14	15
Quality score: number of points/maximum number of points	Sum /11	Sum /12	Sum /14	Sum /15

^a^Gradation: Major = 2, Minor = 1.

^b^Delay from PPH diagnosis.

^c^Measurement of hemoglobin: HemoCue^®^ or red blood count.

^d^Measurement of hemostasis function: ≥ 1 among PT—aPTT—fibrinogen.

*min* minutes, *NA* not applicable.

### Covariables

The characteristics of the maternity units considered were the level of care (level 1, no neonatal unit, to level 3, neonatal intensive care unit), hospital status (public university, other public, private), the annual number of deliveries (< 1000, [1000–2000], [2000–3500], ≥ 3500), and the 24/24 onsite presence of an obstetrician and of an anesthesiologist.

We explored as potential confounders individual covariables including sociodemographic characteristics of the women as well as characteristics of their pregnancy and delivery. We created a PPH risk profile composite variable for the women with at least one risk factor, among a history of PPH, a multiple pregnancy, pre-eclampsia, and delivering a baby of birth weight above 4000 g.

### Analysis

We first described the characteristics of the women and of the maternity units where they gave birth, and then the prevalence of each criterion of quality of care as well as the overall distribution of the quality of care score.

The associations between the characteristics of the maternity units and inadequate care were studied by univariate and then multivariate multilevel logistic regression with a random intercept for the maternity unit. The choice of variables included in the multivariate models was guided by the available literature and by the results of the univariate analysis.

A first model included all the maternal variables selected. Then, we built separate models to estimate the association with quality of care for each hospital characteristic, adjusted for maternal characteristics. We assessed the relevance of combining several of these hospital characteristics in the same model, as those characteristics are very much interrelated. We did so based on the joint distribution of those characteristics in our sample and on the estimation of collinearity of these variables through the calculation of variance Inflation Factors (VIF).

We did a secondary analysis with stratification according to the mode of delivery, i.e. separately among the cases of PPH that occurred after vaginal delivery and among those that happened after cesarean delivery, because we hypothesized that the influence of the organizational environment on the quality of care may vary according to the mode of delivery. This analysis followed the same strategy as the analysis of the whole population.

The proportion of women with at least one missing value in the final multivariate model was 9.6%, principally for the variable “country of birth”. Characteristics of the women with full data were similar to those with missing data, which supported the missing at random hypothesis. We used multiple imputation chained equations according to Rubin’s rules to impute missing data (12 data sets imputed). The results of the univariate and multivariate analyses are presented with the imputed data. We also performed an analysis with the non-imputed data.

The statistical analyses were performed using Stata^®^ software version 15.1. The threshold of significance was set at 0.05.

### Accordance statement

The study was approved by the National Data Protection Authority (Commission Nationale de l’Informatique et des Libertés [CNIL] authorization no. 912210, Mar. 14, 2012). All methods were performed in accordance with the relevant guidelines and regulations. The study methods have been performed in accordance with the Declaration of Helsinki. All women included gave their written consents for the use of their medical records.

### Ethical approval

All women included in this study gave their consents for the use of their medical records.

## Results

Among the 1104 women with severe PPH included in the study, 364 (33%) presented very severe PPH qualified as a “near miss”. No PPH-related death occurred. There were 710 (64.3%) vaginal deliveries and 394 (35.7%) cesarean sections. In 771 (69.8%) of the cases of severe PPH, the cause was uterine atony. The description of the population is given in Table 2.

**Table 2:** Characteristics of women, pregnancy and maternity units among women with severe PPH, and proportion of inadequate care.

Characteristics of women	n (column %^a^)	Proportion with inadequate care^b^ n (row %^c^)	*p*^||^
All women	1104 (100.0)	275 (24.9)	
**Age** **(year)**			
< 25	164 (14.9)	37 (22.6)	
25–35	703 (63.7)	175 (24.9)	
> 35	237 (21.4)	63 (26.6)	
**Country** **of** **birth**			
France	714 (70.9)	184 (25.8)	*
Other European countries	34 (3.4)	8 (23.5)	
North Africa	97 (9.6)	19 (19.6)	
Sub-Saharan Africa	94 (9.3)	12 (12.8)	
Other	68 (6.8)	18 (26.5)	
**BMI** **before** **pregnancy**			
< 18.5	77 (7.2)	19 (24.7)	
[18.5–25]	640 (60.3)	155 (24.2)	
[25–30]	209 (19.7)	54 (25.8)	
≥ 40	136 (12.8)	33 (24.3)	
**Parity**			
Primiparous	549 (50.1)	143 (26.0)	*
Multiparous without previous cesarean	395 (36.0)	83 (21.0)	
Multiparous with previous cesarean	152 (13.9)	45 (29.6)	
**Previous** **PPH**			
Yes	70 (6.3)	13 (18.6)	
No	1034 (93.7)	262 (25.3)	
**Multiple** **pregnancy**			
Yes	114 (10.3)	23 (20.2)	
No	990 (89.7)	252 (25.5)	
**Pre-eclampsia**			
Yes	50 (4.5)	10 (20.0)	
No	1054 (95.5)	265 (25.1)	
**Pre-partum** **hemoglobin ≤ 9** **g/dL**			
Yes	18 (1.7)	6 (33.3)	
No	1037 (98.3)	252 (24.3)	
**Gestational** **age** **at** **delivery** **(weeks)**			
< 37	135 (12.3)	28 (20.7)	
≥ 37	967 (87.7)	246 (25.4)	
**Mode** **of** **delivery**			
Spontaneous vaginal	502 (45.5)	119 (23.7)	
Operative vaginal	208 (18.8)	58 (27.9)	
Antepartum cesarean	201 (18.2)	41 (20.4)	
Intrapartum cesarean	193 (17.5)	57 (29.5)	
**Birth** **weight ≥ 4000** **g**			
Yes	153 (13.9)	35 (22.9)	
No	948 (86.1)	238 (25.1)	
**Woman** **at** **risk** **of** **PPH**^**d**^			
Yes	351 (31.9)	76 (21.7)	*
No	750 (68.1)	197 (26.3)	
**Characteristics** **of** **delivery** **hospitals**			
**Status**			
Public university	446 (40.4)	103 (23,1)	
Public non-university	496 (44.9)	122 (24.6)	
Private	162 (14.7)	50 (30.9)	
**Level** **of** **care**			
3	386 (35.0)	84 (21.8)	**
2	487 (44.1)	117 (24.0)	
1	231 (20.9)	74 (32.0)	
**Annual** **number** **of** **deliveries**			
≥ 3500	200 (18.1)	43 (21.5)	
[2000–3500]	470 (42.6)	108 (23.0)	
[1000–2000]	288 (26.1)	79 (27.4)	
≤ 1000	146 (13.2)	45 (30.8)	
**24/24** **onsite** **presence** **of** **an** **obstetrician-gynecologist**			
Yes	847 (78.1)	205 (24.2)	
No	237 (21.9)	68 (28.7)	
**24/24** **onsite** **presence** **of** **an** **anesthesiologist**			
Yes	989 (91.2)	242 (24.5)	*
No	95 (8.8)	31 (32.6)	

*BMI* body mass index.

^||^p-value for the test of the difference in the % of inadequate care: * ≤ 0.1, ** ≤ 0.05.

^a^Proportion of women with this characteristic in the study population (column %).

^b^Proportion of inadequate care for PPH among women with this characteristic (row %).

^c^Score ≤ 25th percentile of the distribution in the study population.

^d^At least one risk factor among: previous PPH, multiple pregnancy, pre-eclampsia, birth weight ≥ 4000 g.

**Figure 1:**
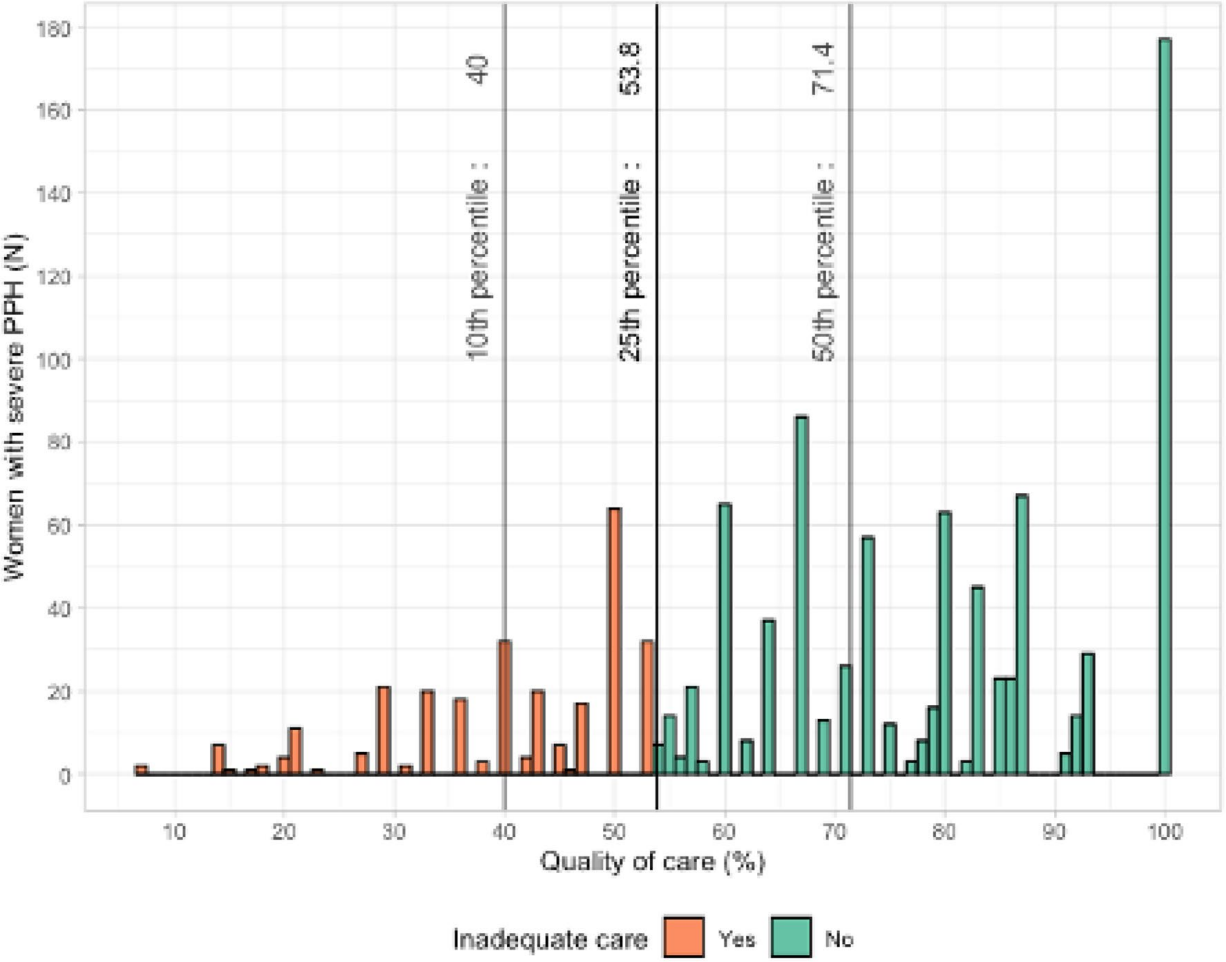
Distribution of the quality of care score among women with severe PPH.

The proportion of compliant management varied from 91.7 to 35.7% depending on the item considered among the 8 selected (Table 3). With the composite score combining these different items, the overall care was fully adequate, i.e. the quality of care score was 100%, in 16% of the women. Twenty-five percent of the women received less than 53.8% of the expected quality of care items (i.e. 25th percentile of the distribution of the score defining inadequate care for the rest of the analysis) (distribution of the score in the study population in Fig. 1).

**Table 3:** Proportion of women with each individual criterion of quality of care for PPH.

Expected item of adequate care	n/N (%)
Prophylactic uterotonic in the third stage of labor	1012/1104 (91.7)
Postpartum blood loss estimation written in the medical chart	937/1104 (84.9)
First-line uterotonic	
*Administered whatever the delay*	982/1104 (88.9)
*Administered ≤ 30 min after PPH diagnosis*	798/1104 (72.3)
Measurement of blood hemoglobin (HemoCue^®^ or red blood cell count) ≤ 60 min after PPH diagnosis	740/1104 (67.0)
Measurement of hemostasis parameters (≥ 1 among PT—aPTT—fibrinogen) ≤ 60 min after PPH diagnosis	508/1104 (46.0)
Manual examination of the uterus ≤ 30 min after PPH diagnosis, in PPH after vaginal delivery	516/710 (72.7)
Examination of the cervix and vagina, in PPH after vaginal delivery	447/710 (63.0)
Second-line uterotonic (sulprostone)	
*Administered whatever the delay, in PPH without uterine atony*	119/333 (35.7)
*Administration ≤ 30 min after the administration of a first line uterotonic (oxytocin) in PPH with uterine atony*	401/771 (52.0)

In the multivariate analysis, the only individual variable significantly associated with a decrease in the risk of inadequate care was maternal birthplace in sub-Saharan Africa (Appendix S5). After taking into account the maternal characteristics, all modes of delivery taken together, the only maternity unit characteristic associated with an increased risk of inadequate care was delivery in a type 1 maternity unit (aOR 1.68 [1.09–2.57] compared with a type 3 maternity unit) (Appendix S6).

Among women with PPH after vaginal delivery, delivery in a type 1 maternity unit was associated with a significant increase in the risk of inadequate care compared with a type 3 maternity unit (aOR 2.04 [1.24–3.35]), as was delivery in a private compared with a public university maternity hospital (aOR 1.72 [1.00–2.97]) or in a maternity unit with fewer than 2000 deliveries per year, compared with maternity units with more than 3500 deliveries per year (< 1000: aOR 2.20 [1.12–4.32]; 1000–2000: aOR 1.90 [1.02–3.56]) (Table 4). Among women with PPH after cesarean delivery, the only variable associated with a significant increase in the risk of inadequate care was delivery in a maternity unit where there was no 24/24 onsite anesthesiologist (aOR 4.34 [1.41–13.31]) (Table 5). The analysis with complete cases showed similar results (results not shown).

**Table 4:** Characteristics of maternity units associated with inadequate care for PPH in women with vaginal deliveries.

	cOR (95% CI)	Model 1	Model 2	Model 3	Model 4	Model 5	Model 6
		Status	Number of deliveries per year	Level of care	Onsite presence of gynecologist	Onsite presence of anesthesiologist	Status and number of deliveries
		aOR (95% CI)	aOR (95% CI)	aOR (95% CI)	aOR (95% CI)	aOR (95% CI)	aOR (95% CI)
**Status**							
Public university	1	1	–	–	–	–	1
Public non-university	1.30 [0.83–2.03]	1.32 [0.85–2.03]	–	–	–	–	1.07 [0.66–1.73]
Private	1.81 [1.04–3.16]	1.72 [1.00–2.97]	–	–	–	–	1.43 [0.82–2.50]
**Number** **of** **deliveries** **per** **year**							
≥ 3500	1	–	1	–	–	–	1
[2000–3500[	1.42 [0.75–2.71]	–	1.50 [0.80–2.80]	–	–	–	1.47 [0.79–2.73]
[1000–2000[	1.98 [1.04–3.77]	–	1.90 [1.02–3.56]	–	–	–	1.76 [0.91–3.37]
< 1000	2.38 [1.20–4.74]	–	2.20 [1.12–4.32]	–	–	–	2.08 [1.02–4.24]
**Level** **of** **care**							
3	1	–	–	1	–	–	–
2	1.36 [0.85–2.16]	–	–	1.39 [0.87–2.20]	–	–	–
1	2.21 [1.33–3.65]	–	–	2.04 [1.24–3.35]	–	–	–
**No 24-h on-site presence of an obstetrician-gynecologist**	1.32 [0.87–2.01]	–	–	–	1.19 [0.78–1.82]	–	–
**No 24-h on-site presence of an anesthesiologist**	1.15 [0.64–2.06]	–	–	–	–	1.02 [0.57–1.82]	–

N = 710 women with severe PPH.

Multilevel logistic regression models (random intercept for maternity unit) with multiple imputation.

Five models, each for 1 delivery hospital characteristic mentioned in the corresponding column, adjusted for individual characteristics (women at risk of PPH (i.e. at least one risk factor among: previous PPH, multiple pregnancy, pre-eclampsia, birth weight ≥ 4000 g), maternal country of birth, parity/previous cesarean delivery).

*CI* confidence interval, *cOR* crude odds ratio, *aOR* adjusted odds ratio.

**Table 5:** Characteristics of maternity units associated with inadequate care for PPH in women with cesarean deliveries.

	cOR (95% CI)	Model 1	Model 2	Model 3	Model 4	Model 5
		Status	Number of deliveries per year	Level of care	Onsite presence of gynecologist	Onsite presence of anesthesiologist
		aOR (95% CI)	aOR (95% CI)	aOR (95% CI)	aOR (95% CI)	aOR (95% CI)
**Status**						
Public university	1	1	–	–	–	–
Public non-university	0.94 [0.52–1.69]	0.88 [0.49–1.60]	–	–	–	–
Private	1.19 [0.53–2.67]	1.12 [0.50–2.53]	–	–	–	–
**Number** **of** **deliveries** **per** **year**						
≥ 3500	1	–	1	–	–	–
[2000–3500[	0.94 [0.45–1.98]	–	0.96 [0.46–2.01]	–	–	–
[1000–2000[	1.09 [0.45–2.63]	–	1.06 [0.44–2.57]	–	–	–
< 1000	1.07 [0.35–3.26]	–	0.99 [0.32–3.07]	–	–	–
**Level** **of** **care**						
3	1	–	–	1	–	––
2	1.01 [0.56–1.84]	–	–	1.01 [0.55–1.84]	–	–
1	1.18 [0.52–2.68]	–	–	1.16 [0.51–2.67]	–	–
**No 24-h on-site presence of an obstetrician-gynecologist**	1.22 [0.59–2.52]	–	–	–	1.20 [0.57–2.51]	–
**No 24-h on-site presence of an anesthesiologist**	4.59 [1.52–13.82]	–	–	–	–	4.34 [1.41–13.31]

N = 394 women with severe PPH.

Multilevel logistic regression models (random intercept for maternity unit) with multiple imputation.

Five models, each for 1 delivery hospital characteristic mentioned in the corresponding column, adjusted for individual characteristics (women at risk of PPH (i.e. at least one risk factor among: previous PPH, multiple pregnancy, pre-eclampsia, birth weight ≥ 4000 g), maternal country of birth, parity/previous cesarean delivery).

*CI* confidence interval, *cOR* crude odds ratio, *aOR* adjusted odds ratio.

## Discussion

### Main findings

We developed a new synthetic assessment tool to quantify the global quality of care for PPH and applied it to a population-based sample of women with severe PPH. While the proportion of adequate care varied widely when considering each item of care separately, the use of our score allowed for an assessment of the global quality of care for PPH. This approach enabled us to explore the determinants of inadequate care and thus identify concrete areas for improvement. We found that some characteristics of the maternity units were associated with an increased risk of inadequate care and that these determinants differed by mode of delivery. Among the women with severe PPH after vaginal delivery, an increased risk of inadequate care was associated with delivery in a type 1 maternity unit, a private maternity unit, or a maternity unit with fewer than 2000 deliveries per year. Among the women with severe PPH after cesarean delivery, the only determinant associated with an increased risk of inadequate care was delivery in a maternity unit where there was no 24/24 onsite presence of an anesthesiologist.

### Strengths and limitations

The main strength of our study was the creation and use of a composite score to evaluate the global quality of care for PPH. This score incorporates several dimensions of care and takes into account specificities related to the mode of delivery and to the etiology of PPH. This score is quantitative and easily reproducible so it can be used to evaluate practices and for monitoring, with a view to assessing the quality of care and disparities in quality over time or between maternity units.

A group of experts selected beforehand the components of care considered from French guidelines, which are very similar to international guidelines^33^. We chose to exclude some particular cases of PPH management (surgical wound without uterine atony, abnormal placental insertion, uterine rupture etc.) in which proper management of PPH require gesture specific to each etiology and circumstance, which are not standardized enough to be relevantly integrated in our score. This strategy allowed us to focus on a population in which PPH guidelines was duly applicable and in which our score was particularly appropriate.

The prospective identification of cases of severe PPH according to a standardized definition enabled exhaustive detection of cases in a source population comprising approximately one-fifth of all deliveries in France in a year. This strengthens the external validity of our results.

To study the determinants of inadequacy, we chose to perform the same analyses according to the mode of delivery, which was made possible by the construction of a quality of care score adaptable to each context. This approach seemed relevant to us because certain practices recommended in the case of PPH differ, thus raising questions regarding different aspects of the organization of care. The results in each of the two strata and the messages were in the end different.

In terms of the limitations of our study, we had to exclude 129 women, i.e. 10% of the population analyzed, because criteria of the score components were missing. However, there were few differences between the women excluded and the women included, which limited the impact of this potential selection bias. For the choice of quality of care criteria, some information on care provided was unavailable and so could not be included in the score, despite the wishes of the experts, notably the time taken to call the different health care professionals and their presence at the time of PPH, which are factors associated with the severity of PPH^5^. However, the fact that all the criteria of quality of care selected correspond to information easily available in the medical charts facilitates the practical use of the quality of care score. Lastly, the possibility of a measurement bias linked to the collection of data from medical charts cannot be eliminated, because a procedure performed but not noted or incompletely noted in the medical charts was considered as not done. However, failure to report clearly in the medical charts procedures that are deemed indispensable by the experts was considered to constitute inadequate care.

### Interpretation

We found that an increased risk of inadequate care for PPH after vaginal delivery was associated with delivery in a private maternity unit, in a maternity unit with a low volume of deliveries, or in a type 1 (low level of care) maternity unit. In France, type 1 maternity units are generally private and small, while type 3 maternity units are usually public, associated with a university hospital, and large^34^. The risk factors for inadequate care revealed in our study therefore often correspond to the same institutions in which the overall care environment seems unfavorable to the proper application of guidelines. A first hypothesis is that the medical teams in institutions with a low volume of activity are less often faced with cases of severe PPH and so are less experienced in their management. Likewise, type 1 maternity units receive patients of low obstetrical risk and their medical teams are less used to managing complex obstetrical situations requiring coordination and team work. The culture of teamwork, particularly through the development of non-technical skills, is an important factor in the improvement of the quality of care for PPH, as shown by educational simulation training works^35,36^. In private maternity units, medical decisions concerning childbirth are taken individually by the referring obstetrician, which predisposes to variability of practices. Our results from real-life observations complete and strengthen those of a previous study which found more self-reported inadequate care in the management of the threat of premature delivery among practitioners from private maternity units^26^. In this study, the principal reported barriers to the application of guidelines were failure to question practitioners’ habits and poor team dialogue. A care environment that favors teamwork and harmonization of practices therefore seems to be important in reducing inadequate care for PPH. For instance, participation in a daily team meeting enabling discussion and debriefing regarding the previous day’s medical charts, as well as reviews of morbidity and mortality, help spread a culture of the evaluation of practices and of continuing training. These kinds of meetings are less frequent in type 1 maternity units and small maternity units^34^ which could therefore explain some of the disparities observed between maternity units^11^.

Finally, it is interesting to note that the determinants of inadequate care identified in our study align with those found in older population-based studies analyzing a specific item of PPH care or a subjective expert assessment^22,23^, but also with the determinants associated with the occurrence of severe complications of PPH^9,37^. These results strengthen the hypothesis of a continuum between inadequate care and worsening of PPH^5^. The use of our score to quantify the relationship between inadequate care and the severity of PPH could constitute an interesting avenue of research to support this hypothesis.

We found that, in PPH after cesarean, the only identified determinant of inadequate care was giving birth in a maternity unit where the anesthesiologist was not present on site 24/24. These results underscore the key role of the anesthesiologist in the management of PPH, as has been reported in several studies^22,37^. Because this factor was only identified in PPH after cesarean section, the post-surgical context seems particularly important in the pathway leading to inadequate care. One hypothesis is that in maternity units where the anesthesiologist is present onsite 24/24, post-cesarean monitoring is done in the recovery room and so is conducted by dedicated staff familiar with the post-operative context. This heightened monitoring could improve early detection of post-operative blood loss and so limit delays to care. Furthermore, the presence of a dedicated anesthesia team in the maternity unit facilitates collaboration between obstetricians and anesthesiologists by strengthening their involvement, for instance, in the implementation of protocols or in other aspects of the internal organization of care, like daily team meeting or reviews of morbidity and mortality. These organizational features, moreover, have been incorporated into multifaceted approaches to the implementation of guidelines on PPH care^38,39^ as well as into the “safety bundle” proposed by the American College of Obstetricians and Gynecologists^17,40,41^. Our results provide further support for their pivotal role in improving the quality of care.

In our study, the only maternal characteristic associated with a decrease in the risk of inadequate care was being born in sub-Saharan Africa. A first hypothesis is that these women are particularly represented in certain maternity units where, for reasons not explained by the variables studied in our models, the risk of inadequate care was reduced. In the French setting, migrant women often give birth in large urban public maternity units^42^. Another hypothesis is that medical teams are more attentive to these women, given their higher risk of severe obstetrical complications^43^.

### Implications

Our results have implications for clinical practice as they provide an assessment of the quality of care for PPH and of the scope for improvement in this field. They are particularly relevant for the categories of maternity units shown to be at higher risk of inadequate care. Dissemination of these results may help local teams to involve in quality-of-care processes, and health care quality agencies to help them in this goal. Our score could be used to monitor quality of care in the same unit over time and between units.

Our results may guide future interventional research by identifying maternity units where opportunities for improvement are the greatest, which are privileged places to test the effectiveness of quality-of-care improvement interventions such as simulation training.

Finally, our results may also be of interest for health care users who look for more transparency on quality of care.

## Conclusion

The development of a new quantitative assessment tool to quantify the global quality of care for PPH allowed the identification of some characteristics of maternity units associated with inadequate care, which indicate where opportunities for improvement are the greatest and where to focus practice improvement initiatives. The quality score developed in this study could be used to monitor the impact of such initiatives.

## Supplementary Information

Supplementary Information.

## Data Availability

The authors confirm that all data used in this study is available upon request from the Editorial Board Members.
