# Supplementary material for: Evaluating the quality of care for postpartum hemorrhage with a new quantitative tool: a population-based study
Source: Sci Rep. 2022 Nov 3;12:18626. doi: 10.1038/s41598-022-23201-0 (PMC9633766; doi:10.1038/s41598-022-23201-0)
Supplement: Supplementary file 1 — Supplementary Information. [file 41598_2022_23201_MOESM1_ESM.docx]

**Appendices**

Appendix S1: EPIMOMS study group

Appendix S2: EPIMOMS definition of Severe Maternal Morbidity (from 22 weeks of gestation until 42 days postpartum)

Appendix S3: Characteristics of women included (N = 1104) and of women not included in the analysis (N = 129)

Appendix S4: Flow chart

Appendix S5: Details of associations between maternal characteristic covariables included in the multivariable models and inadequate care

Appendix S6: Characteristics of maternity units associated with inadequate care for PPH in the total study population

Appendix S1: EPIMOMS study group

For the Naitre en Alsace perinatal network: Bruno LANGER

For the Rhône-Alpes Aurore perinatal network: Corinne DUPONT, René-Charles RUDIGOZ

For the Auvergne perinatal network: Françoise VENDITTELLI

For the Basse-Normandie perinatal network: Gaël BEUCHER

For the MYPA perinatal region, Ile de France region: Patrick ROZENBERG

For the Naitre dans l’Est Francilien network, Ile de France region: Lionel CARBILLON

For the Paris Nord perinatal network: Elie AZRIA, Nathalie BAUNOT

For the 92 Nord perinatal network, Ile de France region: Catherine CRENN-HEBERT, Gilles KAYEM

For the Lorraine perinatal network: Jeanne FRESSON

For the Société Française d’Anesthésie Réanimation (SFAR) : Alexandre MIGNON

For the Santé Publique pole, Hospices Civils de Lyon : Sandrine TOUZET

For the Inserm Epopé research team Marie-Pierre BONNET, Marie-Hélène BOUVIER-COLLE, Anne CHANTRY, Coralie CHIESA-DUBRUILLE, Catherine DENEUX-THARAUX, Aurélien SECO.

Appendix S2: The EPIMOMS definition of Severe Acute Maternal Morbidity (from 22 weeks of gestation until 42 days postpartum)

| **SAMM item** | **Criteria** |
| --- | --- |
| **Major obstetric bleeding** | Blood loss ≥ 1500 mL; and/or hemorrhage with blood transfusion ≥ 4 RBC or arterial embolization or vascular ligation or compressive uterine suture or emergency peripartum hysterectomy or any organ dysfunction (see below) |
| **Eclampsia** | Seizures in a woman diagnosed with pre-eclampsia and, if not, not attributable to another cause |
| **HELLP syndrome** | HELLP syndrome* associated with hepatic hematoma or rupture or with any organ dysfunction (see below)  *Hemolysis, elevated liver enzymes > 3 times the normal level and low platelets < 50 000 |
| **Severe pre-eclampsia** | Pre-eclampsia** if it induced a preterm delivery for a main maternal indication before 32 weeks of gestation or if associated with any organ dysfunction (see below)  ** Defined as a blood pressure ≥ 140/90 and proteinuria ≥ 0.3 g/24 h |
| **Pulmonary embolism** | Clinical symptoms consistent with pulmonary embolism + confirmation with imaging + treatment (imaging: computed tomography or ventilation/perfusion scintigraphy or Doppler; treatment: heparin or thrombolysis or embolectomy) |
| **Stroke** | Cerebral imaging showing cerebral infarction or hemorrhage, or venous thrombosis, or sub-arachnoid hemorrhage |
| **Severe psychiatric disorder** | Severe acute psychiatric disorder or acute decompensation of chronic psychiatric disease (psychosis, major depression, bipolar disorder) diagnosed by psychiatrist and/or suicide attempt |
| **Cardiovascular dysfunction** | Cardiac arrest; and/or acute pulmonary edema with hypoxemia < 60 mmHg or SaO2 < 90% or treated with diuretics; and /or systolic blood pressure < 90 mmHg during > 60 min or not responsive to plasma expansion; and/or acute left ventricular dysfunction: left ventricular ejection fraction < 40% and /or need for continuous IV vasopressor/ inotropic drugs; and/or decompensation of a pre-existing cardiopathy with the need for specialized management; and /or blood troponin > 1 µg/L |
| **Respiratory dysfunction** | Acute hypoxemia < 60 mmHg; or SaO2 < 90% with spontaneous ventilation; and/or mechanical ventilation, or non-invasive ventilation in the absence of chronic disease, not related to anesthesia |
| **Renal dysfunction** | Acute renal failure with blood creatinine > 135 µmol/L  And/or oliguria < 500 mL/24 h |
| **Neurological dysfunction** | Coma, whatever stage and duration |
| **Hepatic dysfunction** | Prothrombin time < 60%, in the absence of constitutional deficiency; and/or bilirubin direct test > 20 mmol/L |
| **Hematological dysfunction** | Acute anemia < 7 g/dL; and/or acute thrombocytopenia < 50 000/mm^3^ in the absence of chronic disease; and/or disseminated intravascular coagulation: platelets < 50 000/mm^3^ or prothrombin time < 60% or fibrinogen < 2 g/L |
| **Emergency surgery apart from delivery procedure** | Secondary hysterectomy; and/or laparotomy for post-delivery complication apart from isolated hematoma or parietal infection |
| **Admission to Intensive Care Unit** | / |
| **Death** | / |
|  |  |

Appendix S3: Characteristics of women included (N = 1104) and of women not included in the analysis (N = 129)

|  | | Women included  N =1104  n (column %) | Women not included  N = 129  n (column %) |
| --- | --- | --- | --- |
| Country of birth | France | 714 (70.9) | 69 (62.7) |
|  | Other European country | 34 (3.4) | 3 (2.7) |
|  | North Africa | 97 (9.6) | 15 (13.6) |
|  | Sub-Saharan Africa | 94 (9.3) | 17 (15.5) |
|  | Other country | 68 (6.8) | 6 (5.5) |
| Parity | Primiparous | 549 (50.1) | 79 (65.9) |
|  | Multiparous without previous cesarean | 395 (36.0) | 28 (23.3) |
|  | Multiparous with previous cesarean | 152 (13.9) | 13 (10.8) |
| Mode of delivery | Spontaneous vaginal | 502 (45.5) | 54 (43.9) |
|  | Operative vaginal | 208 (18.8) | 30 (24.4) |
|  | Antepartum cesarean | 201 (18.2) | 19 (15.4) |
|  | Intrapartum cesarean | 193 (17.5) | 20 (16.3) |
| Women at risk of PPH | Yes | 351 (31.9) | 31 (27.2) |
| Status | Public university | 446 (40.4) | 56 (43.4) |
|  | Public non-university | 496 (44.9) | 59 (45.7) |
|  | Private | 162 (14.7) | 14 (10.9) |
| Level of care | Level 3 | 386 (35.0) | 33 (25.6) |
|  | Level 2 | 487 (44.1) | 73 (56.6) |
|  | Level 1 | 231 (20.9) | 23 (17.8) |
| Number of deliveries per year | ≥ 3500 | 200 (18.1) | 17 (13.2) |
|  | [2000-3500[ | 470 (42.6) | 69 (53.5) |
|  | [1000-2000[ | 288 (26.1) | 17 (13.2) |
|  | < 1000 | 146 (13.2) | 26 (20.1) |
| 24h-hour on site presence of a gynecologist | Yes | 847 (78.1) | 100 (80.6) |
| 24h-hour on site presence of an anesthesiologist | Yes | 989 (91.2) | 118 (95.2) |

Appendix S4: Flow chart


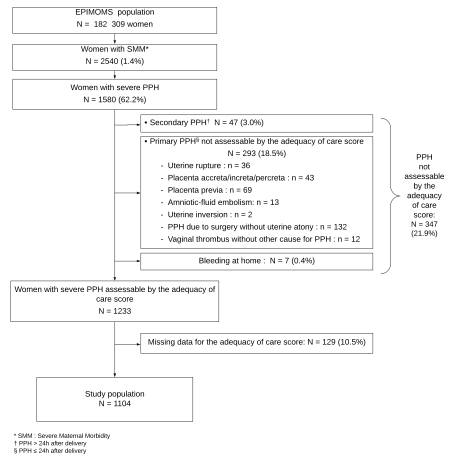


Appendix S5 - Details of associations between maternal characteristic covariables included in the multivariable models and inadequate care

|  |  | Model 1 | Model 2 | Model 3 | Model 4 | Model 5 |
| --- | --- | --- | --- | --- | --- | --- |
|  |  | Status | Number of deliveries per year | Level of care | Onsite presence of gynecologist | Onsite presence of anesthesiologist |
|  | cOR (95% CI) | aOR (95% CI) | aOR (95% CI) | aOR (95% CI) | aOR (95% CI) | aOR (95% CI) |
| Women not at risk of PPH | 1.27 [0.93–1.73] | 1.22 [0.89–1.67] | 1.20 [0.87–1.64] | 1.19 [0.87–1.63] | 1.21 [0.88–1.66] | 1.21 [0.89–1.66] |
| Maternal country of birth |  |  |  |  |  |  |
| France | 1 | 1 | 1 | 1 | 1 | 1 |
| Other Europe country | 1.00 [0.45–2.22] | 0.99 [0.44–2.22] | 0.99 [0.43–2.27] | 1.00 [0.44–2.29] | 1.00 [0.44–2.27] | 1.00 [0.44–2.28] |
| North Africa | 0.72 [0.41–1.27] | 0.73 [0.42–1.27] | 0.75 [0.43–1.30] | 0.74 [0.43–1.28] | 0.74 [0.42–1.29] | 0.73 [0.42–1.27] |
| Sub-Saharan Africa | 0.45 [0.24–0.85] | 0.44 [0.23–0.83] | 0.46 [0.24–0.87] | 0.45 [0.23–0.85] | 0.45 [0.23–0.85] | 0.44 [0.23–0.84] |
| Other country | 1.06 [0.58–1.92] | 1.02 [0.56–1.86] | 1.08 [0.60–1.95] | 1.04 [0.58–1.88] | 1.02 [0.56–1.87] | 1.01 [0.55–1.86] |
| Parity |  |  |  |  |  |  |
| Primiparous | 1 | 1 | 1 | 1 | 1 | 1 |
| Multiparous without previous cesarean | 0.78 [0.57–1.06] | 0.81 [0.59–1.12] | 0.81 [0.59–1.12] | 0.81 [0.59–1.11] | 0.80 [0.58–1.10] | 0.80 [0.58–1.10] |
| Multiparous with previous cesarean | 1.24 [0.82–1.86] | 1.36 [0.90–2.11] | 1.39 [0.92–2.10] | 1.39 [0.92–2.11] | 1.40 [0.92–2.12] | 1.40 [0.92–2.12] |

N = 1104 women with severe PPH.

Multilevel logistic regression models (random intercept for maternity unit) with multiple imputation.

Five models, each for 1 delivery hospital characteristic mentioned in the corresponding column title, including the individual characteristics listed; odds ratios for delivery hospital characteristics are shown in Table 3.

Abbreviations: CI, confidence interval; cOR, crude odds ratio; aOR, adjusted odds ratio

Appendix S6- Characteristics of maternity units associated with inadequate care for PPH in the total study population

|  |  | Model 1 | Model 2 | Model 3 | Model 4 | Model 5 |
| --- | --- | --- | --- | --- | --- | --- |
|  | cOR (95% CI) | Status | Number of deliveries per year | Level of care | Onsite presence of gynecologist | Onsite presence of anesthesiologist |
|  |  | aOR (95% CI) | aOR (95% CI) | aOR (95% CI) | aOR (95% CI) | aOR (95% CI) |
| Status |  |  |  |  |  |  |
| Public university | 1 | 1 | / | / | / | / |
| Public non-university | 1.14 [0.79–1.65] | 1.13 [0.79–1.61] | / | / | / | / |
| Private | 1.56 [0.98–2.49] | 1.48 [0.94–2.33] | / | / | / | / |
| Number of deliveries per year |  |  |  |  |  |  |
| ≥ 3500 | 1 | / | 1 | / | / | / |
| [2000-3500[ | 1.15 [0.68–1.94] | / | 1.16 [0.70–1.92] | / | / | / |
| [1000-2000[ | 1.52 [0.88–2.63] | / | 1.46 [0.86–2.47] | / | / | / |
| < 1000 | 1.77 [0.97–3.21] | / | 1.63 [0.91–2.93] | / | / | / |
| Level of care |  |  |  |  |  |  |
| 3 | 1 | / | / | 1 | / | / |
| 2 | 1.17 [0.79–1.72] | / | / | 1.16 [0.80–1.70] | / | / |
| 1 | 1.78 [1.16–2.75] | / | / | 1.68 [1.09–2.57] | / | / |
| No 24-hour on-site presence of an obstetrician-gynecologist | 1.29 [0.89–1.86] | / | / | / | 1.21 [0.84 –1.74] | / |
| No 24-hour on-site presence of an anesthesiologist | 1.49 [0.90–2.47] | / | / | / | / | 1.39 [0.84 –2.28] |

N = 1104 women with severe PPH.

Multilevel logistic regression models (random intercept for maternity unit) with multiple imputation.

Five models, each for 1 delivery hospital characteristic mentioned in the corresponding column, adjusted for individual characteristics (women at risk of PPH (i.e. at least one risk factor among: previous PPH, multiple pregnancy, pre-eclampsia, birth weight ≥ 4000 g), maternal country of birth, parity/previous cesarean delivery); odds ratios for individual characteristics are shown in Appendix S6.

Abbreviations: CI, confidence interval; cOR, crude odds ratio; aOR, adjusted odds r
